# Use and application of geographical restrictions in systematic reviews with the aim of including studies about Germany: An update of a methodological review

**DOI:** 10.1111/hir.12555

**Published:** 2024-12-05

**Authors:** Catharina Muente, Alexander Pachanov, Julian Hirt, Falk Hoffmann, Rebecca Palm, Silvan Munschek, Dawid Pieper

**Affiliations:** ^1^ Faculty of Health Sciences Brandenburg, Brandenburg Medical School (Theodor Fontane) Institute for Health Services and Health System Research Rüdersdorf Germany; ^2^ Center for Health Services Research Brandenburg Medical School (Theodor Fontane) Rüdersdorf Germany; ^3^ Pragmatic Evidence Lab, Research Center for Clinical Neuroimmunology and Neuroscience Basel (RC2NB) University Hospital Basel and University of Basel Basel Switzerland; ^4^ Institute of Health and Nursing Science, Medical Faculty Martin Luther University Halle‐Wittenberg Halle (Saale) Germany; ^5^ Department of Health Eastern Switzerland University of Applied Sciences St. Gallen Switzerland; ^6^ Department of Health Services Research, Faculty VI Medicine and Health Sciences Carl von Ossietzky University Oldenburg Oldenburg Germany; ^7^ School of Nursing Science, Faculty of Health, Department of Nursing Science Witten/Herdecke University Witten Germany

**Keywords:** database searching, Europe, central, indexing, literature searching, recall, review, systematic, review, systematic ‐ as topic, search strategies

## Abstract

**Background:**

In systematic reviews (SRs), geographical limitations in literature searches can aid in focussing research efforts. A methodological review published in 2016 examined the approaches SR authors use to identify studies about Germany, analysing 36 SRs.

**Objective:**

The aim of this study was to update the original review.

**Methods:**

We conducted a literature search on PubMed for SRs synthesising evidence from studies about Germany published between 22 January 2016 and 7 June 2022. Two reviewers independently performed study selection and data extraction. We evaluated the application of search syntax for restricting studies to those about Germany using the peer review of electronic search strategies criteria. The updated findings were reported and summarised alongside those of the original review.

**Results:**

Thirty‐two additional SRs were newly included (total = 68). Geographic restrictions were applied in 57 SRs, representing 72% in the original review and increasing to 97% in the newly included SRs. Moreover, there was an increased use of truncations and field tags.

**Conclusion:**

Although geographical restriction methods are increasingly utilised, additional tools are necessary to enhance the robustness of search strategies. The development of a dedicated geographical search filter would facilitate the identification of studies about Germany.

**Key Messages**

- Currently applied search methods are not sufficient for a highly sensitive search to identify studies about Germany.
- Developing a geographical search filter would aid in identifying studies about Germany.
- A search filter designed to identify studies about Germany should incorporate the truncation ‘German*’ and include city names in both German and English within the author's affiliations field tag.

## BACKGROUND

Systematic reviews (SRs) are crucial for evidence based healthcare and decision making. SRs aim to identify, evaluate and summarise the available evidence to answer a specific research question. They are a compressed form of knowledge accumulation and are consulted for information on current healthcare relevant questions or as a basis for evidence based decisions in health care (Aromataris & Pearson, 2014); therefore, they must be of high quality. When SRs emerged, they initially focused mainly on the effects of healthcare interventions, often including only randomised controlled trials (RCTs) (Clarke, 2016; Moher et al., 2007).

In these cases, geographic restrictions have almost no impact on the results.

However, in the past 20 years, the focus has shifted beyond the effectiveness of healthcare interventions to areas such as aetiology, prevalence and diagnosis (Hoffmann et al., 2021). As the range of SRs has broadened, encompassing various methodologies and topics, the geographical context of the research has become increasingly important. Thus, limiting literature searches enables researchers to conduct reviews in a resource‐efficient and effective manner. A well defined and documented search strategy is a vital part of an SR (Aromataris & Munn, 2020). Integrating geographic restrictions into the search strategy may be useful for several reasons.

1. **Cultural insights:** Geographic restrictions can provide valuable insights into how culture affects health outcomes and healthcare delivery. They can also offer valuable insights into the interrelationships among culture, health outcomes and health delivery (Gadebusch Bondio et al., 2020; Michalski et al., 2017).
2. **Epidemiological analysis:** By focussing on specific geographical regions, researchers can analyse the prevalence, incidence and distribution of diseases and health conditions within these areas. This provides a more nuanced understanding of the epidemiological patterns and facilitates targeted interventions (Aumann, Prenzler, et al., 2014; Rapp et al., 2019).
3. **Health economics:** Geographic restrictions allow researchers to explore economic factors that influence healthcare in a particular region. This includes assessing healthcare expenditure, resource allocation and the cost‐effectiveness of interventions, which can vary significantly across geographic areas (Eden et al., 2021; Kirsch, Teuner, et al., 2013).
4. **Comparison between different geographical areas:** Geographic restrictions facilitate comparisons between different healthcare settings or strategies across distinct regional areas, allowing for a comparative analysis that can highlight variations in healthcare delivery models and their respective effects on patient outcomes, thereby guiding decision making and best practices (Badia et al., 2017; Laffet et al., 2021)
5. **Differences and inequalities within a specific region:** Examining healthcare within a specific region allows for the identification and analysis of differences and inequalities in care provision, access and quality (Klein & Knesebeck, 2018; Schneider et al., 2014).

From a methodological perspective, limiting a literature search to a specific geographical area poses challenges for both researchers and librarians because it requires thorough knowledge of the relevant geographical terms, regional characteristics and possible linguistic differences.

However, several analyses indicate that search strategies in general are often inadequate (Faggion et al., 2013; Sampson & McGowan, 2006). For instance, in MEDLINE, common search errors include missing or irrelevant Medical Subject Heading (MeSH) terms, unwarranted explosion of MeSH terms or missing spelling variants (Sampson & McGowan, 2006). An inadequate search strategy can exclude relevant studies from a review, leading to biased conclusions.

However, little is known about how authors of SRs proceed when they need to geographically restrict their literature search. A previous methodological review conducted in 2016 addressed how authors limited their literature searches in SRs when only studies from Germany were included (Pieper et al., 2016). Examining how authors of SRs proceed will allow us to compile the available approaches to geographical restrictions in the literature and their frequency of use. This exploration also provides an opportunity to discuss the application of these methods and how geographic restrictions can be enhanced.

Limiting the literature searches for conducting SRs to those about Germany facilitates that the research findings directly address the specificities of the German healthcare system, making them more culturally and contextually embedded. It also offers valuable insights into the healthcare policies, interventions and outcomes specific to Germany. In addition, geographical limitation to studies about Germany enables comparative analyses with studies from other countries or regions.

The authors of the original methodological review, published in 2016, found that search strategies using geographic restrictions are often insufficiently developed. Thus, there is a risk that relevant studies will be overlooked (Pieper et al., 2016).

Garner et al. (2016) recommended that updates to SRs may be necessary if the research question remains relevant and new studies have been published that could affect the results of the evidence synthesis. Considering that ~80 new systematic reviews are published every day, and this number continues to increase (Hoffmann et al., 2021), these conditions are met. Additionally, it is recommended that when evaluating the findings of a particular systematic review, users should search for more recent reviews or studies to determine whether new evidence is available and whether the results are consistent with those of the previous review (Shojania et al., 2007).

The aims of this methodological review were twofold: first, we aimed to update the previous review and analyse how the authors currently proceed in their literature searches when only studies in Germany are to be included. We analysed the differences in the search syntax used by the authors of the included SRs between the original review and the update. Second, this update will serve as preliminary work for the development of a search filter to identify studies in Germany using the relative recall technique (Sampson et al., 2006). This approach involves creating a gold standard set from the records included in evidence syntheses relevant to the search filter being developed (Sampson et al., 2006).

## METHODS

### Protocol and registration

We conducted a methodological review to examine the methodological aspects of SRs and to identify the strengths and weaknesses of the methods used. This review was updated in accordance with a protocol registered in the Open Science Framework <Add OSF Link here>. As there are no reporting guidelines for methodological reviews (Lawson et al., 2020; Mbuagbaw et al., 2020), we followed the Preferred Reporting Items for Systematic Reviews and Meta‐Analyses 2020 checklist (Page et al., 2021) and the Guidelines for Reporting Meta‐Epidemiological Methodology Research (Murad & Wang, 2017), where appropriate.

### Eligibility criteria

Study type: in the original review, publications were considered if they were labelled as SRs and searched for literature using at least one bibliographic database. Occasionally, reviews are incorrectly labelled as SRs (Hoffmann et al., 2021). Therefore, we refined the eligibility criteria following a proposal by Krnic Martinic et al. (2019). This update included reviews reporting the following:

- research question,
- sources that were searched using a reproducible search strategy (naming of databases, naming of search platforms/engines, search date and complete search strategy for at least one information source),
- inclusion and exclusion criteria,
- study selection (screening) methods and
- information about data analysis and synthesis.

If an article labelled as an SR had missing or insufficient information, we first requested details from the corresponding author(s). If the author(s) provided the requested information, we reassessed whether the article met the eligibility criteria. The first or the corresponding author was contacted once to clarify any ambiguities.

Focus: SRs should refer to studies about Germany. If several countries were investigated, the results for Germany should be reported separately. We did not restrict the studies based on the thematic foci.

Language: English or German.

### Information sources

A literature search was conducted on PubMed on 7 June 2022. We considered the reports published from 22 January 2016 onwards, because this was the last search date of the original review. PubMed provides a representative overview of the literature and is typically used in (bio‐)medical, epidemiological and healthcare research. Using PubMed as the primary data source ensured a broad and informed basis for our analyses and evaluations, as over 85% of primary studies included in systematic reviews are indexed on MEDLINE (Booth, 2016), which can be searched via PubMed.

### Search strategy

The search strategy remained the same as that used in 2016, including a search filter for SRs with a combination of the search terms ‘Deutsch*’ or ‘German*’ in the title only. This approach is based on the assumption that titles of SRs usually contain a specific geographical region to emphasise the scope and context of the research. Appendix A presents the complete search strategy.

### Selection process

Two members of the research team independently assessed the titles, abstracts and full texts (<Author1> and <Author2>) for inclusion criteria. Disagreements were resolved through discussion. If no agreement could be reached, a third team member (<Author7>) was asked to make the final decision. In the case of multiple versions of an SR (e.g., updates), only the most recent version was considered. The screening was conducted using a Rayyan web app (Ouzzani et al., 2016).

### Data collection process

Data were extracted by one reviewer (<Author1>) and double‐checked by a second reviewer (<Author2>). Discrepancies were resolved by consensus. The data were extracted using Microsoft Word and the new data were integrated with the data obtained in the original review.

### Data items

We extracted the following data: authors, journal, language in which the SR was written, number of included primary studies, thematic focus of the SR search sources used, geographic restriction of the search syntax, indexing of the SR with ‘Germany’ in PubMed MeSH term and whether ‘Germany’ or ‘Deutschland’ was mentioned in the author address field of any author.

It is of particular interest whether ‘Germany’ or ‘Deutschland’ is mentioned in the affiliations of the SR, because this facilitates the identification and search for relevant SRs. The categories of ‘intervention’, ‘health economics’, ‘epidemiology’ or ‘various’ were selected to assign the thematic focus of the included SR. Analysing thematic focus is crucial to understand which thematic fields and geographical restrictions are used in SRs. The search sources used were categorised into: database search (e.g., MEDLINE), hand search, expert request, reference check, bibliography check of relevant authors, web search, congress abstracts, search in journals, ‘similar articles function’ and own data.

Following the definition of the Cochrane Collaboration, handsearching refers to the manual searching of sources (Higgins et al., 2022). This is distinct from electronic searching, which includes using the search function within Portable Document Format congress proceedings. Web searches include searching through the pages of relevant institutions or organisations (e.g., professional societies and clinics) (Higgins et al., 2022). Searching in journals involves journal archives that were not covered by the searched databases, as noted (Pieper et al., 2016). Reference checking entails reviewing the reference lists of included studies and previous reviews on the same topic (Higgins et al., 2022). Own data refer to primary studies already known to the author group from previous research projects. Utilising the ‘similar articles function’ involves searching for related articles using the corresponding function within a (bibliographic) database (e.g., PubMed).

Regarding the search syntax, we focused on the component related to geographical restriction specific to Germany.

### Analytical approach

No formal assessment of the quality of the included SRs was conducted. Instead, the search syntax for the geographic restrictions of studies about Germany in the included SRs was analysed. We analysed the search syntax with a focus on the geographical restriction to Germany, following the most recently updated Peer Review of Electronic Search Strategies (PRESS) criteria (McGowan et al., 2016). We examined whether an attempt was made to geographically restrict the search syntax to Germany. If this was the case, we addressed the question of how the geographical restrictions were imposed.

The use of truncations, search terms, field tags, MeSH and Emtree are the recommended methods to restrict literature searches (Bramer et al., 2018). Therefore, we analysed these aspects in more detail.

We examined the extent to which truncations of the terms ‘Deutsch*’ and ‘German*’ were used. We also investigated whether other geographic terms (cities and states) were searched, and if field tags were used. Field tags refer to search operators that allow users to specify the fields of a record they want to search. For example, PubMed offers various field tags, such as [ti]: search for the term in the article title (MEDLINE/PubMed Data Element (Field) Descriptions, 2019).

We included any field tags used by the authors of the SRs in order to geographically restrict studies to Germany. In addition, we checked whether the term Germany was searched using MeSH or Emtree.

### Synthesis methods

The results were summarised narratively and combined with the results of the 2016 review. In addition, the results of the original review and this update were compared.

## RESULTS

### Study selection

**FIGURE 1:**
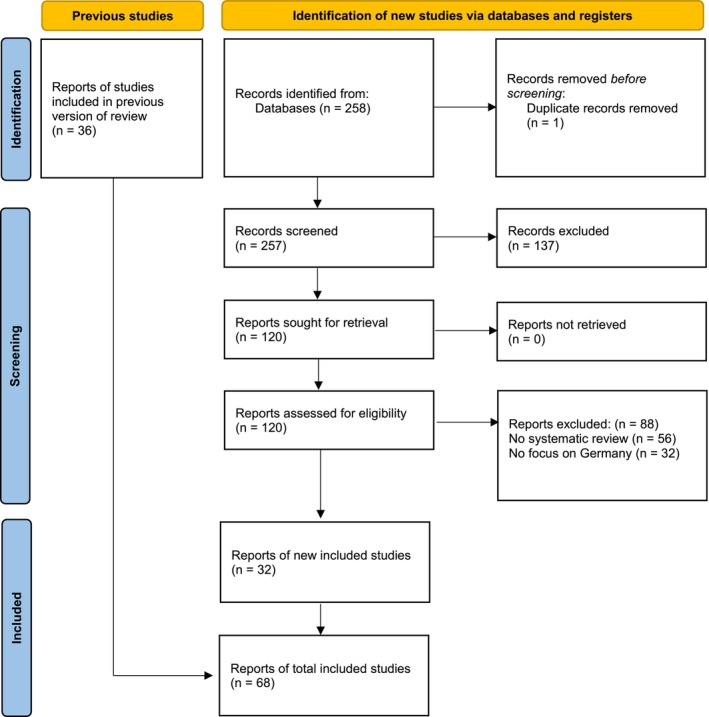
PRISMA 2020 flow diagram of included reports. [Colour figure can be viewed at wileyonlinelibrary.com]

The PubMed search yielded 258 hits, of which 120 reports were classified as potentially relevant and screened at the full‐text level; 32 SRs were newly included. Together with the 36 SRs from the original review, the total number of SRs included was 68. A flowchart illustrates the selection process (Figure 1). In total, 44 authors were contacted owing to insufficient detail in their reported search strategies. Thirty‐two authors responded to our inquiry.

### Study characteristics

The earliest SR was published in 2005, with the most recent one published in 2022. A total of 79.4% (54/68) of the SRs were published in 2013 or later. The number of primary studies included in the SRs ranged from 1 to 435, with a median of 22 (interquartile range [IQR] 37–13). Among the first authors, 10.3% (7/68) were not affiliated with any institutions in Germany; 61.8% (42/68) of the SRs were published in English. The SRs covered various foci: epidemiology (*n* = 27), intervention (*n* = 12), health economics (*n* = 14) and other topics (*n* = 20), with multiple assignments possible. A comprehensive table of the characteristics of the included SRs is presented in Table B1: Appendix B, and Table 1 provides a summarised overview.

**TABLE 1:** Descriptive study characteristics.

	Total (*n*)	Original SR (*n*)	Update (*n*)
Number of included SRs	68	36	32
Number of included reports, total	2271	1019	1252
Number of included reports, range	1–435	1–131	2–435
Median	22	21	24.5
IQR	37–13	47–13	36.5–12

Abbreviations: IQR, Interquartile range; SR, systematic review.

### Search strategies

Various strategies were used to identify the primary studies. The majority of SRs, 86.8% (59/68), used at least two different search sources. The number of databases searched varied from 1 to 11, with a median of 2 (IQR: 1–5). Other search sources were reference checks (*n* = 39), Internet search (*n* = 22), handsearching (*n* = 21), expert request (*n* = 9), journal research (*n* = 4), congress abstracts (*n* = 4), bibliography check of authors (n = 3), own data (*n* = 4) and the ‘similar articles function’ (*n* = 2). Table B1: Appendix B provides an overview of the search strategies used in the included SRs.

### Geographic restrictions

Geographical restrictions for Germany were undertaken in 83.8% (57/68) of the included SRs; 14.7% (10/68) of the SRs were not indexed with the MeSH term ‘Germany’. Truncation of the terms ‘German*’ or ‘Deutsch*’ (*corresponds to truncation) was found in 25% (17/68) of the SRs. The use of any field tags was reported in 33.8% (23/68). The following field tags were used: title (8/68), title/abstract (7/68), abstract (7/68), MESH (7/68), keywords (6/68), expansion (3/68), affiliation (3/68), other terms (2/68), text word (2/68), address (1/68), classification (1/68), full text (1/68) and language (1/68).

Of the SRs, 7.4% (5/68) searched for cities or federal states, whereas 17.7% (12/68) used only the search term ‘Germany’, without the use of further adjectives or other geographic terms such as cities or federal states. Table B1: Appendix B provides the search syntax.

### Comparison of original review and update

When comparing the original review with the data from this update, several trends are evident. Regarding the study characteristics, the following differences can be reported: 91% (29/32) of the newly included SRs were written in English, compared with 36% (13/36) in the original review. However, the affiliation of the authors was more often marked with the term ‘Germany’: 44.4% (16/36) of the original and 87.5% (28/32) of the newly included SRs. At the time of publication of the original review, five of the included SRs were not labelled with the MeSH term ‘Germany’; however, this is currently the case for only two SRs. In this update, eight SRs were not tagged with the MeSH term ‘Germany’, and five of these SRs were published in journals that were not indexed in MEDLINE.

Regarding the search strategy, the following differences can be reported. In numerous SRs, geographic restrictions were used: 72.2% (26/36) in the original SR versus 96.9% (31/32) in the newly included SRs. In the SRs included in the update, at least two databases were used in 81.3% (26/32) of SRs, whereas this was the case in 66.7% (24/36) in the original review. A comparison of the results from the original publication and the update regarding the search strategies applied is presented in Table 2.

**TABLE 2:** Search strategies of included studies.

Search strategies	Total % (*n*)	Original SR % (*n*)	Update % (*n*)
Geographical restriction to Germany	83.8 (57)	72.2 (26)	96.9 (31)
Truncation of the terms German* or Deutsch*	25.0 (17)	8.3 (3)	43.8 (14)
Search for Germany in MeSH	16.2 (11)	8.3 (3)	25.0 (8)
Search for geographical terms (e.g., Cities, federal states)	7.4 (5)	2.8 (1)	12.5 (4)
Search only for Germany	17.7 (12)	28.0 (10)	6.3 (2)
Field tags	33.8 (23)	16.7 (6)	53.1 (17)

Abbreviations: MeSH, Medical Subject Heading; SR, systematic review.

## DISCUSSION

In this updated methodological review, we explored the benefits of geographically restricting literature searches. This approach can aid researchers and librarians in improving their search strategies, particularly for authors of SRs who may not have access to librarian support.

The original review noted that the search syntax for regional restrictions to Germany was not well developed (Pieper et al., 2016). Comparing the original review with the update revealed several notable trends. A substantial portion of the newly included SRs are published in English, underscoring the growing international relevance of studies focused on Germany. Additionally, there is an increase in the number of authors utilising at least two databases for their literature searches.

However, several factors should be considered when interpreting these trends. First, literature related to Germany is sometimes intentionally published in German to target German speaking audiences. Second, gaps exist in the electronic indexing of studies about Germany (Pieper et al., 2016). This issue arises because German‐language articles are occasionally published in journals not indexed in MEDLINE (Blümle & Antes, 2008; Pieper et al., 2016). Third, gaps in electronic indexing were highlighted in a hand search study published in 2008, which examined RCTs and controlled clinical trials (CCTs) across 85 medical journals as part of a Cochrane Collaboration project (Blümle & Antes, 2008). The identified studies were compared with entries in MEDLINE, and the number of journals and identified studies was included in MEDLINE. Among the journals searched, 55% of the German‐language articles were not included in MEDLINE (Blümle & Antes, 2008). Since the original review in 2016, the indexation process in MEDLINE has evolved. As of April 2022, all journals indexed for MEDLINE undergo automated indexing of MeSH terms using the article's title and abstract, supplemented by human review and curation as appropriate. As none of the included SRs conducted their literature search after April 2022, this does not affect this review. However, future studies could investigate if there is a difference in usage of subject headings regarding the geographic restriction to Germany pre‐April 2022 compared to post‐April 2022.

Finally, 64.7% (44/68) of the authors of the included SRs had to be contacted, because their search strategies were not clearly reported. Other studies have also indicated that the reporting of search strategies in SRs is poorly described (Rethlefsen et al., 2023).

In addition to general trends, the efforts to limit geographical literature searches to Germany have increased considerably. Geographical restrictions were reported in considerably more SRs, and numerous authors used additional terms, such as federal states or cities, instead of solely using the term Germany. In addition, the number of SRs using truncated ‘German*’ and ‘Deutsch*’ terms for the search increased. Therefore, there is an ongoing effort to optimise search syntaxes, aligning them more closely with the PRESS guidelines (McGowan et al., 2016) and other recommended practices (Bramer et al., 2018).

However, although these results suggest a more sophisticated search syntax, not even half of the newly included SRs have used ‘German*’ or ‘Deutsch*’ truncation. More SRs are now indexed with the MeSH term ‘Germany’ compared to the original review, although 25% remain unindexed in the update. Indexing SRs with the MeSH term ‘Germany’ is crucial for effective literature searches, ensuring that research findings related to Germany are easily accessible and discoverable.

A similar result emerged for the use of field tags; this implies a tendency to use field tags more frequently. Nevertheless, the proportion was just over 50% even for the newly included SRs. In the original review, the authors discussed that the not frequently utilised field tags to restrict literature searches to Germany do not result in bias in the results of SRs, but they do increase the workload because more hits need to be screened (Pieper et al., 2016). Whether this is owing to lack of knowledge or a deliberate choice, the outcome remains the same—no bias in the results but an increased workload due to the need to check more hits (Pieper et al., 2016).

Despite these trends, there is potential for further improvement in the search syntax used. A filter would aid in facilitating geographically restricted literature searches, as search filters decrease the number of irrelevant hits compared to search strategies without filters (Jenkins, 2004). Geographic search filters with varying degrees of precision and sensitivity already exist for some regions, including Spain (Valderas et al., 2006), Africa (Pienaar et al., 2011), the United Kingdom (Ayiku et al., 2017, 2019; Ayiku, Levay, et al., 2020), the United States (Cheung et al., 2022), German speaking countries (specific for high impact factor nursing journals) (Hirt et al., 2019) and the countries of the Organization for Economic Co‐operation and Development (Ayiku, Hudson, et al., 2021; Ayiku, Levay, et al., 2021). In addition, there is a low‐ and middle‐income countries geographic search filter to identify studies on preterm birth prevention and management (Sutton & Campbell, 2022).

An overview of geographic search filters can be found on the website of the InterTASC Information Specialists' Sub‐Group Search Filter Resource (Glanville et al., 2006). In a scoping review, an overview of topic search filters is already provided (Damarell et al., 2019). Further studies could systematically identify which validated geographical search filters are already available.

We have used the findings of this study to develop a geographical search filter for identifying studies about Germany (Pachanov et al., 2024). For example, we found that nearly 90% of the authors of the included SRs were affiliated with institutions in Germany. We conclude that integrating the ‘affiliation’ field tag would be beneficial for a geographical filter in PubMed. This result aligns with a previous study that recommends adding an additional line to the filter to search for institutions (Sutton & Campbell, 2022).

In addition, the development of the geographical search filter to identify studies about Germany will be based on existing approaches to search filter development (Ayiku, Craven, et al., 2020; Glanville et al., 2008).

### Limitations

This update has particular limitations. It adheres to the SR definition provided by Krnic Martinic et al. (2019), differing from the original review, although this is unlikely to impact reported results. Restricting searches to ‘Deutsch*’ or ‘German*’ in titles alone may not yield comprehensive results. In addition, we did not extend searches to other German place names or organisations to maintain consistency with the original review for comparability, potentially overlooking relevant studies. Only one database was queried, which may have led to important sources being overlooked. We considered only SRs published in German or English, with not all authors responding to queries. Another limitation is that we did not perform a forward–backward search, which could have likely identified additional reports. We chose to omit this step, to maintain consistency with the search strategy used in the original review, thereby enabling a direct comparison. Another limitation of this review is that the literature search was conducted in 2022, which means that recent SRs published since then may not be included in the analysis.

Together, these limitations suggest the possibility of overlooking relevant studies. Furthermore, to facilitate analysis, the complexity of included SRs was simplified. This means that the method described in the SRs may not have been fully observed in the analysis.

## CONCLUSION

This methodological review has shown that more sensitive approaches to geographically restrict literature searches to Germany are being used by the authors of the included SRs compared to the original review.

There were improvements in all the areas we addressed, such as geographical restriction to Germany, truncation of the terms German* or Deutsch*, search for Germany in MeSH, search for geographical terms (e.g., cities and federal states) and usage of field tags. Despite these noticeable improvements over time, the search strategies used remain highly heterogeneous and could benefit from optimisation. Therefore, as highlighted in the original review (Pieper et al., 2016), in addition to following existing guidelines (McGowan et al., 2016) and recommendations (Bramer et al., 2018), there is a clear need for a well structured search syntax that includes advanced tools such as geographic search filters. Our methodological review underscores the necessity for a systematically developed and validated geographical search filter to effectively identify studies about Germany.

## AUTHOR CONTRIBUTIONS

CM, AP, DP and JH were involved in the conception or design of the manuscript. CM and AP reviewed the articles, DP functioned as referee. CM, AP, SM and DP did the analysis and interpretation of the data. CM drafted the manuscript. CM, AP, FH, RP, JH, SM and DP were involved in the critical revision of the manuscript and final approval of the manuscript. The authors read and approved the final manuscript.

## FUNDING INFORMATION

This review is a part of the project ‘Development and validation of a geographic search filter to identify studies conducted in Germany’, which is funded by the German Research Foundation (Deutsche Forschungsgemeinschaft‐DFG; project number 471387773).

## CONFLICT OF INTEREST STATEMENT

The authors declare that they have no competing interests.

## INFORMED CONSENT

All authors involved have read and approved the final manuscript.

## Data Availability

The data that support the findings of this study are openly available in Open Science Framework at https://osf.io/6gt2m.

## APPENDIX A

### A.1 SEARCH STRATEGY

Search conducted in PubMed on 7 June 2022.

((‘Meta‐Analysis’[Publication Type] OR ‘Meta‐Analysis as Topic’[Mesh] OR meta analy*[TIAB] OR metaanaly*[TIAB] OR systematic review*[TIAB] OR ‘systematic literature review*’[TIAB] OR ‘systematic overview*’[TIAB] OR ‘Review Literature as Topic’[Mesh] OR ‘reference list*’[TIAB] OR bibliograph*[TIAB] OR hand‐search*[TIAB] OR ‘relevant journals’[TIAB] OR ‘manual search*’[TIAB] OR ((‘selection criteria’[TIAB] OR ‘inclusion criteria’[TIAB] OR ‘data extraction’[TIAB]) AND (review[Publication Type])))).

NOT (‘Comment’[Publication Type] OR ‘Letter’[Publication Type] OR ‘Editorial’[Publication Type]) AND (english[LA] OR german[LA]).

AND (German*[TI] OR Deutsch*[TI]).

Records retrieved: 258.

## APPENDIX B

### B.1

**TABLE B1:** Comprehensive overview of the study characteristics.

Review	Journal	*N* publications	Focus	Search strategies	Syntax Germany	Tagged with Germany (MeSH)	Author address contains ‘Germany’
Aumann et al. (2014)	Pneumologie	23	Epidemiology Health economics	Database search (*n* = 1) Handsearching	germany OR german	Yes	No
Barkmann and Schulte‐Markwort (2012)	Journal of epidemiology and community health^a^	34	Epidemiology	Database search (*n* = 8) Expert request Handsearching Own data	n.r.^c^	Yes	Yes
Bauer et al. (2021)	Der Anaesthesist	15	Epidemiology	Own data Database search (*n* = 2)	No restriction	Yes	Yes
Becka et al. (2014)	Deutsche medizinische Wochenschrift	52	Other	Database search (*n* = 5) Internet search	‘deutsch’ oder ‘german’	Yes	No
Bittner (2014)	Das Gesundheitswesen	12	Epidemiology	Database search (*n* = 2) Reference check	German OR Germany^c^	Yes	No
Bozorgmehr et al. (2016)	BundesgesundheitsblattGesundheitsforschungGesundheitsschutz	52	Other	Database search (*n* = 11) Internet search Reference check Expert request	german* *‘The search in databases included titles, abstracts and keywords, without any restriction regarding time period or language’ (Author's translation)*	No	Yes
Bozorgmehr et al. (2017)	Euro surveillance^a^	6	Epidemiology	Database search (*n* = 11) Internet search Reference check Expert request	german* *‘The search in databases included titles, abstracts and keywords, without any restriction regarding time period or language’*	Yes	Yes
Buchholz and Kohlmann (2013)	Die rehabilitation	20	Other	Database search (*n* = 4) Research in journals Internet search Congressional abstracts Reference check Bibliography check of authors	No restriction	Yes	No
Castaneda (2012)^b^	Social science & medicine^a^	n.r.	Other	Database search (*n* = 1) Handsearching	Germany (MeSH)	Yes	n.a.
Damm et al. (2016)	International journal of public health^a^	36	Epidemiology Health Economics	Database search (*n* = 2) Reference check	‘Germany’ OR ‘German’ OR ‘Deutschland’ OR ‘deutsche’ OR ‘deutsches’ OR ‘deutscher’ OR ‘deutschen’ OR ‘Berlin’ OR ‘Hamburg’ OR ‘Munich’ OR ‘Munchen’ OR ‘Cologne’ OR ‘Koln’ OR ‘Frankfurt’ OR ‘Stuttgart’ OR ‘Dusseldorf’	Yes	Yes
Dreier et al. (2012)	Das Gesundheitswesen	13	Epidemiology	Database search (*n* = 11) Internet search Research in journals	No restriction	Yes	No
Eden et al. (2021)	Psychiatrische praxis	14	Health economics	Database search (*n* = 2) Reference check	german*^c^	Yes	No
Evans et al. (2012)^b^	Patient education and counseling^a^	32	Epidemiology	Database search (*n* = 11) Reference check Bibliography check of authors Research in journals Expert request	Germany OR German*	Yes	No
Fuchs et al. (2014)	Deutsches Ärzteblatt International^a^	17	Intervention	Database search (*n* = 4) Reference check	Germany[TIAB] OR GERMANY[Mesh] OR Deutschland[TT] OR german[TIAB]	Yes	No
Gansen (2018)	BMC health services research^a^	35	Health economics	Database search (*n* = 2) Handsearching	‘Germany’[MeSH] OR German OR Germany	Yes	Yes
Gräske et al. (2013)	BundesgesundheitsblattGesundheitsforschung Gesundheitsschutz	47	Other	Database search (*n* = 4)	No restriction	Yes	Yes
Gruhn et al. (2022)	Vaccine^a^	36	Health economics epidemiology	Database search (*n* = 2) Reference check	‘germany’ [Title/Abstract] OR ‘german’ [Title/Abstract] OR ‘german’ [Affiliation] OR ‘germany’ [Affiliation] OR ‘deutschland’ [Affiliation]	Yes	Yes
Grupp et al. (2014)	The journal of mental health policy and economics	48	Health economics	Database search (*n* = 1) Reference check	german*	Yes	Yes
Hajak et al. (2021)^b^	Frontiers in psychiatry^a^	13	Other	Database search (*n* = 6) Reference check	‘Germany*’^c^	No, journal not indexed in MEDLINE	Yes
Hajek et al. (2022)	Frontiers in medicine^a^	12	Epidemiology	Database search (*n* = 3) Reference check	German* OR Germany [MeSH Terms]	No, journal not indexed in MEDLINE	Yes
Hansen et al. (2022a) (1)	Journal der Deutschen Dermatologischen Gesellschaft^a^	30	Epidemiology	Database search (*n* = 1) Reference check	German OR Germany ‘*in AllFields*’	Yes	Yes
Hansen et al. (2022c) (2)	Journal der Deutschen Dermatologischen Gesellschaft^a^	37	Epidemiology	Database search (*n* = 1) Reference check	German OR Germany ‘*in AllFields*’	No	Yes
Hansen et al. (2022b) (3)	Journal der Deutschen Dermatologischen Gesellschaft^a^	12	Epidemiology	Database search (*n* = 1) Reference check	German OR Germany ‘*in AllFields*’	Yes	Yes
Heinmüller et al. (2016)	Trials^a^	62	Other	Database search (*n* = 1) Reference check Handsearching internet search	AFFILCOUNTRY (deutschland) OR AFFILCOUNTRY (germany)	Yes	Yes
Heller et al. (2014)	Deutsche medizinische Wochenschrift	10	Epidemiology	Database search (*n* = 1) Congressional abstracts	n.r.^c^	Yes	No
Hendricks et al. (2021)	International journal of surgery^a^	7	Other	Database search (*n* = 2)	(germany OR german)	Yes	Yes
Hoell et al. (2021)	BJPsych open^a^	30	Other	Database search (*n* = 7) Reference check Internet search	Germany [MeSH Term] German* [Text Word]^c^	No, journal not indexed in MEDLINE	Yes
Huppe & Raspe, 2005	Die rehabilitation	16	Intervention	Database search (*n* = 6) Congressional abstracts Expert request	No restriction	Yes	No
Idelevich et al. (2016)	Bundesgesundheitsblatt Gesundheitsforschung Gesundheitsschutz	n.r.	Epidemiology	Database search (*n* = 1) Reference check Handsearching	Germany^c^	Yes	Yes
Kallert et al. (2005)	Das Gesundheitswesen	n.r.	Intervention	Database search (*n* = 3) Own data	Germany^c^	Yes	No
Kauffmann et al. (2020)^b^	Expert review of vaccines^a^	49 (123 in total)	Epidemiology Health economics	Database search (*n* = 3) Internet search Handsearching	German* OR deutsch*	Yes	Yes
Khan and Ollenschlager (2014)	Zeitschrift für Evidenz, Fortbildung und Qualität im Gesundheitswesen.	18	Intervention	Database search (*n* = 2) Internet search Reference check Similar articles function Handsearching	No restriction	Yes	Yes
Kien et al. (2018)^b^	Implementation science^a^	38	Intervention	Database search (*n* = 5) Handsearching Reference check Expert request Related articles function	Germany [Affiliation] OR Deutschland [Affiliation]	Yes	No
Kirsch et al. (2013)	Das Gesundheitswesen	14	Health economics	Database search (*n* = 4)	Germany	Yes	No
Klein and Knesebeck (2018)	International journal for equity in health^a^	63	Other	Database search (*n* = 1) Reference check Handsearching	(german* [TIAB] OR german [language])	Yes	Yes
Kock et al. (2011)^b^	Deutsches Ärzteblatt International^a^	n.r.	Epidemiology	Database search (*n* = 1) Handsearching	Germany^c^	Yes	Yes
Konnopka et al. (2009)	Psychiatrische Praxis	11	Health economics	Database search (*n* = 2) Reference check	No restriction	Yes	No
Korber et al. (2013)	Das Gesundheitswesen	18	Health economics	Database search (*n* = 6) Reference check	Germany	Yes	No
Kornischka et al. (2008)	Psychiatrische Praxis	n.r.	Epidemiology	Database search (*n* = 1) Internet search Handsearching Reference check	German^c^	Yes	No
Kreis et al. (2016)	Health policy^a^	435	Other	Database search (*n* = 3) Handsearching	‘German?’ OR ‘Deutsch?’ ‘*All searches within titles, keywords, and abstracts covered the period from 2000 to 2014*. *Only articles written in German or English were included*.’	Yes	Yes
Liebherz and Rabung (2013)^b^	Psychotherapie, Psychosomatik, medizinische Psychologie	131	Intervention	Database search (*n* = 1) Reference check Internet search	No restriction	Yes	No
Liebherz and Rabung (2014)^b^	PLoS ONE^a^	67	Intervention	Database search (*n* = 1) Handsearching Reference check Internet search	No restriction	Yes	Yes
Linde et al. (2014)	Forschende Komplementärmedizin = Research in complementary medicine	12	Other	Database search (*n* = 1) Internet search Reference check Expert request	Germany [tw] OR German [tw]	Yes	Yes
Löhler et al. (2019)	European archives of oto‐rhino‐laryngology^a^	10	Epidemiology	Database search (*n* = 8) Internet search Reference check Handsearching	exp Germany/ exp. Europe/ (german* or BRD or deutsch*).ti,ab,kf,ot. or europ*.ti,ot. +57 further place or region information	Yes	Yes
Lösch et al. (2022)	Das Gesundheitswesen^a^	16	Other	Database search (*n* = 3) Internet search Handsearching Reference check	Deutschland OR deutsch OR Germany OR german OR ‘Bundesrepublik Deutschland’	Yes	No
Menzler et al. (2019)^b^	Clinical ophthalmology^a^	31	Epidemiology	Database search (*n* = 2) Own data	‘*The resulting set of articles was condensed by searching for the term “German” in all search fields*’.	No, journal not indexed in MEDLINE	Yes
Michelmann and Himmel (2005)	Das Gesundheitswesen	32	Other	Database search (*n* = 1) Handsearching Research in journals	Germany^c^	Yes	No
Miesbach et al. (2019)^b^	Orphanet journal of rare diseases^a^	2	Epidemiology	Database search (*n* = 4) Internet search	Germany	Yes	Yes
Mittag et al. (2011)	European journal of cardiovascular prevention and rehabilitation^a^	77 (Studies)	Intervention	Database search (*n* = 5) Reference check Congressional abstracts Expert request	No restriction	Yes	Yes
Nieder (2012)^b^	Strahlentherapie und Onkologie^a^	106	Other	Database search (*n* = 1) Bibliography check of authors	Germany^c^	Yes	n.a.
Palm et al. (2013)	Pflege	22	Intervention	Database search (*n* = 6) Handsearching Internet search Reference check	Germany OR Deutschland	Yes	No
Pieper et al. (2015)	BMC health services research^a^	19	Other	Database search (*n* = 4)	‘germany’/exp. OR germany:ca,ad,ab,ti OR german:ca,ad,ab,ti OR gkv:ab,ti OR deutschland:ca,ad,ab,ti OR deutsch:ca,ad,ab,ti OR berlin:ca,ad,ab,ti + 138 further place resp. Region information	Yes	Yes
Rapp et al. (2019)	Zeitschrift für Gerontologie und Geriatrie^a^	27	Epidemiology	Database search (*n* = 1) Reference check	Germany [Title/Abstract] OR German [Title/Abstract]	Yes	Yes
Rosner et al. (2019)^b^	PLoS ONE^a^	12	Other	Database search (*n* = 2) Internet search	germany [MeSH] OR german*	Yes	No
Schaller et al. (2021)^a^	PLoS ONE	29	Other	Database search (*n* = 2) Reference check Handsearching	german*	Yes	Yes
Schaller et al. (2022)^a^	BMC nursing	11	Intervention	Database search (*n* = 2) Reference check	german*	No, journal not indexed in MEDLINE	Yes
Schmid (2015)	Health economics review^a^	13	Health economics	Database search (*n* = 5) Reference check	‘German’ OR ‘Germany’ [full text] ‘The terms are searched in title/abstract if there is no additional explanation’	No, journal not indexed in MEDLINE	Yes
Schmucker et al. (2019)^a^	BMC public health	16	Epidemiology	Database search (*n* = 8) Internet search Handsearching	exp Germany/ exp. Europe/ (german* or BRD or deutsch*).ti,ab,kf,ot. or europ*.ti,ot. +57 further place or region information	Yes	Yes
Schneider et al. (2014)	Systematic reviews^a^	n.r.	Intervention	Database search (*n* = 13) Internet search	German* title, abstract, and key words	Yes	Yes
Schulz et al. (2018a) (1)^a^	Journal of public health	25	Other	Database search (*n* = 2)	German* [Title/Abstract]	Yes	Yes
Schulz et al. (2018b) (2)^a^	International journal of environmental health research	18	Other	Database search (*n* = 4)	German* [Title/Abstract]	Yes	Yes
Steffen et al. (2021)^a^	BMC infectious diseases	68	Epidemiology	Database search (n = 3) Handsearching	germany [MESH] OR german* [Title/Abstract] OR german* [Text Word] +20 further place or region information in [Title/Abstract] and [Text Word]	No	Yes
Stegbauer et al. (2020)^b^	BMC health services research^a^	16 (24 in total)	Health economics	Database search (*n* = 3)	german germany	Yes	Yes
Stratmann et al. (2007)	Der Schmerz	22	Health economics	Database search (*n* = 11)	‘In addition, the search was limited to data for Germany.’^c^, ^d^	Yes	Yes
Taubner et al. (2013)^b^	Praxis der Kinderpsychologie und Kinderpsychiatrie	11	Intervention	Database search (*n* = 6) Internet search Reference check Expert request	‘Deutschland’ oder ‘Germany’^c^	Yes	n.a.
Tubbicke et al. (2012)	European journal of clinical microbiology & infectious diseases^a^	87	Epidemiology Health economics	Database search (*n* = 2) Reference check	Germany OR German [each in longer expressions, such as ‘prevalence German hospitals’]^c^	Yes	Yes
Walzer et al. (2013)^b^	ClinicoEconomics and outcomes research^a^	1	Epidemiology	Database search (*n* = 2) Reference check	No restriction	No, journal not indexed in MEDLINE	Yes
Weissenborn et al. (2015)	Das Gesundheitswesen	35	Epidemiology	Database search (*n* = 2) Reference check Internet search	Germany	Yes	Yes

Abbreviations: MeSH, Medical Subheading; *n*, Number; n.a., not applicable; n.r., not reported.

^a^ English language.

^b^ Coauthors based outside Germany.

^c^ Search syntax is not (completely) available or cannot be reconstructed (regarding syntax Germany).

^d^ Translated by authors.
